# 5*H*-Imidazo[4,5-*f*][1,10]phenanthroline

**DOI:** 10.1107/S1600536812016595

**Published:** 2012-04-21

**Authors:** Shao-Wei Tong, Wen-Dong Song, Dong-Liang Miao, Jing-Bo An

**Affiliations:** a College of Food Science and Technology, Guangdong Ocean University, Zhanjiang 524088, People’s Republic of China; b College of Science, Guangdong Ocean University, Zhanjiang 524088, People’s Republic of China

## Abstract

The title mol­ecule, C_13_H_8_N_4_, is is essentially planar [r.m.s. deviation for all non-H atoms = 0.025 (3) Å]. In the crystal, mol­ecules are connected through one weak bifurcated N—H⋯(N,N) hydrogen bond and three π–π stacking inter­actions between pyridine and imidazole rings [centroid–centroid distance = 3.631 (8) Å] and between pyridine and benzene rings [centroid–centroid distances = 3.675 (5) and 3.666 (2) Å].

## Related literature

For our previous work based on 1,10-phenanthroline as anauxiliary ligand, see: Song *et al.* (2009 ▶); Hao *et al.* (2008 ▶). For background to 1,10-phenanthroline complexes, see: Chesnut *et al.* (1999 ▶).
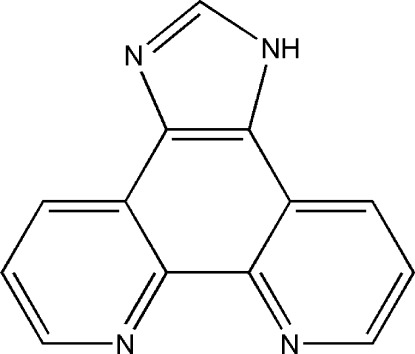


## Experimental

### 

#### Crystal data

C_13_H_8_N_4_
*M* *_r_* = 220.23
Orthorhombic, 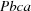

*a* = 14.569 (2) Å
*b* = 7.8623 (12) Å
*c* = 17.042 (3) Å
*V* = 1952.1 (5) Å^3^
*Z* = 8
Mo *K*α radiation
μ = 0.10 mm^−1^
*T* = 296 K
0.26 × 0.18 × 0.16 mm

#### Data collection

Bruker APEXII CCD diffractometer
Absorption correction: multi-scan (*SADABS*; Bruker, 2007 ▶) *T*_min_ = 0.980, *T*_max_ = 0.985
5772 measured reflections
1756 independent reflections
1025 reflections with *I* > 2σ(*I*)
*R*_int_ = 0.053

#### Refinement

*R*[*F* ^2^ > 2σ(*F* ^2^)] = 0.042
*wR*(*F* ^2^) = 0.123
*S* = 1.00
1756 reflections
186 parameters
All H-atom parameters refined
Δρ_max_ = 0.18 e Å^−3^
Δρ_min_ = −0.15 e Å^−3^

### 

Data collection: *APEX2* (Bruker, 2007 ▶); cell refinement: *SAINT* (Bruker, 2007 ▶); data reduction: *SAINT*; program(s) used to solve structure: *SHELXS97* (Sheldrick, 2008 ▶); program(s) used to refine structure: *SHELXL97* (Sheldrick, 2008 ▶); molecular graphics: *SHELXTL* (Sheldrick, 2008 ▶); software used to prepare material for publication: *SHELXTL*.

## Supplementary Material

Crystal structure: contains datablock(s) I, global. DOI: 10.1107/S1600536812016595/bx2404sup1.cif

Structure factors: contains datablock(s) I. DOI: 10.1107/S1600536812016595/bx2404Isup2.hkl

Supplementary material file. DOI: 10.1107/S1600536812016595/bx2404Isup3.cml

Additional supplementary materials:  crystallographic information; 3D view; checkCIF report

## Figures and Tables

**Table 1:** Hydrogen-bond geometry (Å, °)

*D*—H⋯*A*	*D*—H	H⋯*A*	*D*⋯*A*	*D*—H⋯*A*
N3—H8⋯N1^i^	0.92 (3)	2.47 (3)	3.104 (3)	126 (2)
N3—H8⋯N2^i^	0.92 (3)	2.18 (3)	3.017 (3)	150 (2)

Symmetry code: (i) 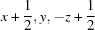.

**Fig. 1.**
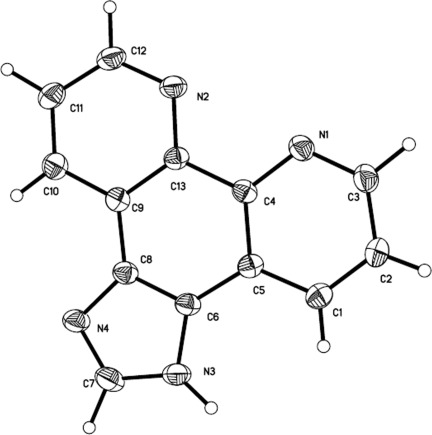
Molecular configuration and atom numbering scheme for the title complex showing 30% probability ellipsoids.

**Fig. 2.**
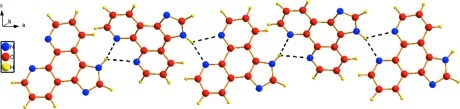
Part of the crystal structure showing one dimensional chain along (100).

## supplementary crystallographic information

### Comment

(1,10)phenanthroline is widely used to synthetize metal-complex as a auxiliary ligand. In our laboratory earlier studies, we have successfully obtained some metal-complex with (1,10)phenanthroline.We report here the crystal structure of the title compound, Fig. 1. The title compound, is essentially planar (r.m.s. deviation for all non-H atoms = 0.025 (3)Å). In the crystal the molecules are connected through one weak bifurcated N—H···N hydrogen bonds, Fig. 2, Table 1 and π-π stacking interactions between pyridine and imidazole rings [distance centroid – centroid 3.631 (8) Å] and between pyridine and benzene rings [distance centroid–centroid 3.675 (5); 3.666 (2) Å] respectively.

### Experimental

Imidazo(4,5-f)(1,10)phenanthroline was purchased from Jinan Henghua science and technology limited company and recristallized of an alkaline solution.

### Refinement

All H-atoms in this structure were located from differences Fourier syntheses and were refined isotropically.

### Crystal data

**Table:**

C_13_H_8_N_4_	*F*(000) = 912
*M**_r_* = 220.23	*D*_x_ = 1.499 Mg m^−^^3^
Orthorhombic, *P**b**c**a*	Mo *K*α radiation, λ = 0.71073 Å
Hall symbol: -P 2ac 2ab	Cell parameters from 799 reflections
*a* = 14.569 (2) Å	θ = 2.2–27.5°
*b* = 7.8623 (12) Å	µ = 0.10 mm^−^^1^
*c* = 17.042 (3) Å	*T* = 296 K
*V* = 1952.1 (5) Å^3^	Block, yellow
*Z* = 8	0.26 × 0.18 × 0.16 mm

### Data collection

**Table:**

Bruker APEXII CCD diffractometer	1756 independent reflections
Radiation source: fine-focus sealed tube	1025 reflections with *I* > 2σ(*I*)
Graphite monochromator	*R*_int_ = 0.053
φ and ω scans	θ_max_ = 25.2°, θ_min_ = 2.4°
Absorption correction: multi-scan (*SADABS*; Bruker, 2007)	*h* = −17→10
*T*_min_ = 0.980, *T*_max_ = 0.985	*k* = −9→9
5772 measured reflections	*l* = −20→20

### Refinement

**Table:**

Refinement on *F*^2^	Primary atom site location: structure-invariant direct methods
Least-squares matrix: full	Secondary atom site location: difference Fourier map
*R*[*F*^2^ > 2σ(*F*^2^)] = 0.042	Hydrogen site location: inferred from neighbouring sites
*wR*(*F*^2^) = 0.123	All H-atom parameters refined
*S* = 1.00	*w* = 1/[σ^2^(*F*_o_^2^) + (0.0606*P*)^2^] where *P* = (*F*_o_^2^ + 2*F*_c_^2^)/3
1756 reflections	(Δ/σ)_max_ < 0.001
186 parameters	Δρ_max_ = 0.18 e Å^−^^3^
0 restraints	Δρ_min_ = −0.15 e Å^−^^3^

### Special details

**Table:**

Geometry. All esds (except the esd in the dihedral angle between two l.s. planes) are estimated using the full covariance matrix. The cell esds are taken into account individually in the estimation of esds in distances, angles and torsion angles; correlations between esds in cell parameters are only used when they are defined by crystal symmetry. An approximate (isotropic) treatment of cell esds is used for estimating esds involving l.s. planes.
Refinement. Refinement of F^2^ against ALL reflections. The weighted R-factor wR and goodness of fit S are based on F^2^, conventional R-factors R are based on F, with F set to zero for negative F^2^. The threshold expression of F^2^ > 2sigma(F^2^) is used only for calculating R-factors(gt) etc. and is not relevant to the choice of reflections for refinement. R-factors based on F^2^ are statistically about twice as large as those based on F, and R- factors based on ALL data will be even larger.

### Fractional atomic coordinates and isotropic or equivalent isotropic displacement parameters (Å^2^)

**Table:**

	*x*	*y*	*z*	*U*_iso_*/*U*_eq_	
N2	0.08583 (13)	0.0741 (3)	0.18218 (11)	0.0428 (6)	
C13	0.17857 (16)	0.0868 (3)	0.18200 (12)	0.0349 (6)	
C4	0.22247 (15)	0.1620 (3)	0.25102 (12)	0.0347 (6)	
N1	0.16716 (13)	0.2161 (3)	0.30974 (10)	0.0423 (6)	
C5	0.31923 (15)	0.1740 (3)	0.25518 (12)	0.0355 (6)	
N3	0.46339 (14)	0.1051 (3)	0.17574 (13)	0.0471 (6)	
N4	0.39502 (14)	−0.0018 (3)	0.06803 (11)	0.0449 (6)	
C11	0.09449 (19)	−0.0623 (4)	0.05615 (15)	0.0487 (7)	
C12	0.04717 (19)	−0.0004 (3)	0.12071 (16)	0.0494 (7)	
C3	0.20665 (19)	0.2840 (3)	0.37251 (14)	0.0460 (7)	
C2	0.30087 (18)	0.3045 (3)	0.38092 (14)	0.0439 (7)	
C1	0.35746 (19)	0.2504 (3)	0.32252 (13)	0.0416 (6)	
C6	0.37040 (15)	0.1101 (3)	0.18996 (12)	0.0365 (6)	
C7	0.4727 (2)	0.0392 (3)	0.10225 (15)	0.0514 (7)	
C8	0.32970 (16)	0.0437 (3)	0.12418 (12)	0.0373 (6)	
C9	0.23250 (15)	0.0280 (3)	0.11878 (12)	0.0356 (6)	
C10	0.18762 (19)	−0.0478 (3)	0.05489 (14)	0.0445 (7)	
H7	0.5396 (16)	0.027 (3)	0.0759 (12)	0.042 (6)*	
H6	0.2264 (16)	−0.088 (3)	0.0121 (15)	0.056 (8)*	
H4	−0.0192 (17)	−0.012 (3)	0.1232 (13)	0.045 (7)*	
H1	0.4230 (16)	0.270 (3)	0.3229 (13)	0.051 (8)*	
H5	0.0590 (16)	−0.125 (3)	0.0140 (15)	0.063 (8)*	
H3	0.1640 (15)	0.322 (3)	0.4153 (13)	0.046 (7)*	
H2	0.3235 (16)	0.353 (3)	0.4266 (14)	0.053 (7)*	
H8	0.5057 (19)	0.136 (4)	0.2135 (18)	0.074 (9)*	

### Atomic displacement parameters (Å^2^)

**Table:**

	*U*^11^	*U*^22^	*U*^33^	*U*^12^	*U*^13^	*U*^23^
N2	0.0304 (12)	0.0574 (14)	0.0407 (12)	−0.0012 (10)	−0.0020 (9)	0.0005 (10)
C13	0.0326 (13)	0.0403 (15)	0.0318 (12)	−0.0009 (11)	−0.0029 (11)	0.0052 (10)
C4	0.0343 (13)	0.0379 (14)	0.0319 (11)	0.0024 (12)	0.0035 (11)	0.0054 (10)
N1	0.0396 (12)	0.0519 (14)	0.0355 (11)	0.0021 (10)	0.0037 (10)	0.0001 (9)
C5	0.0350 (14)	0.0389 (14)	0.0326 (12)	−0.0016 (11)	−0.0027 (11)	0.0075 (10)
N3	0.0308 (12)	0.0644 (15)	0.0462 (13)	0.0046 (11)	−0.0024 (11)	0.0001 (11)
N4	0.0334 (12)	0.0646 (15)	0.0368 (11)	0.0021 (11)	0.0034 (10)	0.0000 (10)
C11	0.0435 (17)	0.0584 (18)	0.0441 (15)	−0.0023 (14)	−0.0125 (13)	−0.0028 (13)
C12	0.0326 (15)	0.0651 (19)	0.0505 (15)	−0.0029 (14)	−0.0084 (13)	−0.0022 (13)
C3	0.0488 (17)	0.0546 (18)	0.0346 (14)	0.0041 (13)	0.0018 (12)	−0.0029 (11)
C2	0.0511 (17)	0.0462 (16)	0.0344 (13)	−0.0027 (13)	−0.0045 (12)	−0.0036 (11)
C1	0.0403 (15)	0.0443 (15)	0.0403 (14)	−0.0014 (13)	−0.0053 (13)	0.0029 (11)
C6	0.0305 (13)	0.0445 (15)	0.0344 (12)	0.0013 (11)	0.0015 (10)	0.0047 (11)
C7	0.0401 (17)	0.0647 (19)	0.0495 (15)	0.0122 (15)	0.0068 (14)	0.0010 (13)
C8	0.0335 (14)	0.0447 (16)	0.0338 (12)	0.0021 (12)	0.0013 (11)	0.0039 (10)
C9	0.0375 (14)	0.0372 (14)	0.0321 (12)	0.0020 (11)	−0.0006 (10)	0.0048 (10)
C10	0.0471 (17)	0.0518 (17)	0.0346 (13)	0.0026 (14)	−0.0025 (12)	0.0009 (11)

### Geometric parameters (Å, º)

**Table:**

N2—C12	1.326 (3)	C11—C10	1.362 (4)
N2—C13	1.355 (3)	C11—C12	1.387 (4)
C13—C9	1.411 (3)	C11—H5	1.01 (3)
C13—C4	1.464 (3)	C12—H4	0.97 (2)
C4—N1	1.353 (3)	C3—C2	1.390 (4)
C4—C5	1.415 (3)	C3—H3	1.00 (2)
N1—C3	1.327 (3)	C2—C1	1.360 (3)
C5—C1	1.410 (3)	C2—H2	0.93 (2)
C5—C6	1.429 (3)	C1—H1	0.97 (2)
N3—C7	1.362 (3)	C6—C8	1.372 (3)
N3—C6	1.377 (3)	C7—H7	1.08 (2)
N3—H8	0.92 (3)	C8—C9	1.424 (3)
N4—C7	1.314 (3)	C9—C10	1.403 (3)
N4—C8	1.396 (3)	C10—H6	0.98 (3)
			
C12—N2—C13	117.0 (2)	C2—C3—H3	120.2 (13)
N2—C13—C9	122.2 (2)	C1—C2—C3	119.2 (2)
N2—C13—C4	117.63 (19)	C1—C2—H2	121.8 (15)
C9—C13—C4	120.2 (2)	C3—C2—H2	119.0 (15)
N1—C4—C5	122.4 (2)	C2—C1—C5	119.3 (2)
N1—C4—C13	117.4 (2)	C2—C1—H1	123.0 (14)
C5—C4—C13	120.16 (19)	C5—C1—H1	117.5 (14)
C3—N1—C4	117.7 (2)	C8—C6—N3	105.7 (2)
C1—C5—C4	117.6 (2)	C8—C6—C5	122.9 (2)
C1—C5—C6	125.2 (2)	N3—C6—C5	131.3 (2)
C4—C5—C6	117.2 (2)	N4—C7—N3	114.5 (2)
C7—N3—C6	105.7 (2)	N4—C7—H7	124.9 (11)
C7—N3—H8	132.4 (17)	N3—C7—H7	120.5 (11)
C6—N3—H8	121.7 (17)	C6—C8—N4	111.3 (2)
C7—N4—C8	102.7 (2)	C6—C8—C9	121.0 (2)
C10—C11—C12	118.6 (2)	N4—C8—C9	127.7 (2)
C10—C11—H5	122.6 (13)	C10—C9—C13	118.2 (2)
C12—C11—H5	118.7 (13)	C10—C9—C8	123.4 (2)
N2—C12—C11	124.8 (2)	C13—C9—C8	118.4 (2)
N2—C12—H4	115.4 (14)	C11—C10—C9	119.2 (2)
C11—C12—H4	119.7 (14)	C11—C10—H6	124.2 (14)
N1—C3—C2	123.9 (2)	C9—C10—H6	116.6 (14)
N1—C3—H3	115.9 (13)		

### Hydrogen-bond geometry (Å, º)

**Table:**

*D*—H···*A*	*D*—H	H···*A*	*D*···*A*	*D*—H···*A*
N3—H8···N1^i^	0.92 (3)	2.47 (3)	3.104 (3)	126 (2)
N3—H8···N2^i^	0.92 (3)	2.18 (3)	3.017 (3)	150 (2)

Symmetry code: (i) *x*+1/2, *y*, −*z*+1/2.
